# Personal

**Published:** 1916-11

**Authors:** 


### PERSONAL

---

Announcement of removal, travel, and other matters of interest are requested. Please report errors in the listing of any physician in the State and other directories, that we may co-operate with the State Society in securing a correct list.

---

Dr. A. L. Benedict of Buffalo calls attention to a mistake in his name in the classified list (physicians) in the 1916 Buffalo City Directory and requests those using this list to correct as above, the initials being the full name.

---

Dr. E. W. Kennedy announces his return to Rochester. Office 53 S. Fitzhugh Street, residence 127 Genesee Street. Both telephone connections at both places. Practice limited to diseases of the eye; office hours by appointment.

---

Dr. Roland O. Meisenbach of Buffalo spent Aug. and Sept. in the east.

---

Dr. J. E. Slotkin of Buffalo has moved from 537 William Street to 49 Niagara Street.

Dr. Frank A. Valente of Buffalo, a member of the Hospital Corps of the 74th Reg. was home on furlough during part of Oct. and Nov.

Dr. Clayton W. Greene of Buffalo, 1st Lt. U. S. Medical Reserve Corps, who has been in charge of the depot hospital at Columbus, was granted a 4-weeks leave of absence, during Oct. and Nov.

Our Associate Editor, Henry W. Hill, LL. D., has been re-elected president of the N. Y. State Waterways Assn.

Drs. Wm. G. Bissell and Geo. W. Diebold of the Buffalo Health Dept. made a tour of the state laboratories in Oct. with special reference to the study of infantile paralysis.

Dr. Herman G. Matzinger returned in Oct. from a motor tour of the east.

Drs. Herman K. De Groat, Arthur E. Eisbein, Jesse N. Roe and Geo. H. Westinghouse have qualified by examination for appointment as city diagnostician at a salary of $2000.

Dr. Sophy Ellen Page, Buffalo 1902, married Rev. Charles L. Carlucci of Monson, a suburb of Springfield, Mass., Oct. 5.

Dr. F. W. Maeder of Albany, representing the state health dept. made an inspection of Tonawanda and adjacent towns about Oct. 1, to study the typhoid situation.

Dr. A. C. Callahan of Buffalo, of the hospital staff of the 74th Reg., had a furlough in Oct. He reports the men of the regiment as enjoying the best health in spite of bad weather.

Dr. Rolla E. Shaver, Buffalo and Dr. Grace A. Joslin of E. Bethany, graduates of the Medical Dept. of the University of Buffalo, 1915, were married in Buffalo, Oct. 14. After a wedding trip in the west, they will reside at 1225 Clinton Street, Buffalo.

Dr. Allen A. Jones of Buffalo gave a dinner at the Saturn Club, Oct. 19, of 20 covers, in honor of the out-of-town guests of the Medical Assn. of Central N. Y.

Dr. Lucien Howe of Buffalo entertained the members and guests of the Medical Assn. of Central N. Y. at luncheon, Oct. 19. Col. J. R. Kean, U. S. A. spoke briefly of the organization of Red Cross Units.

